# A Provocative Molecular Link between Mammographic Density and BRCA1-loss associated TNBC

**DOI:** 10.18689/ijhg-1000101

**Published:** 2019-06-21

**Authors:** Jingyao Xu, Gbinigie Olusola, Alexus Footman, Nora Hansen, Aswathy Miriam Cheriyan, Krishna Koganti, Vaishali Reddy, Samir Yezdani, Vikram Eddy, Henry De’smond, Nicolas Bakinde, Joel Okoli, Gabriela Oprea, Kathleen Gundry, E Shyam P Reddy, Veena N Rao

**Affiliations:** 1 Cancer Biology Program, Department of OB/GYN, Morehouse School of Medicine, Georgia Cancer Center for Excellence, Grady Health System, Atlanta, USA; 2 Depatment of Internal Medicine, Morehouse School of Medicine, Georgia Cancer Center for Excellence, Grady Health System, Atlanta, USA; 3 Department of Surgery, Morehouse School of Medicine, Georgia Cancer Center for Excellence, Grady Health System, Atlanta, USA; 4 Department of Pathology, Emory University Hospital, Atlanta, USA

**Keywords:** BRCA1, Mammographic density, Ubc9, TNBC, Collagen, Fibronectin, SIRT1, ER-α, β-Catenin, BAT, WAT

## Abstract

Triple-negative breast cancer (TNBC) is a highly aggressive form of breast cancer that has a high mortality rate and disproportionately affects young African American (AA) women who carry mutations in the BRCA1 gene. Approximately 80% of breast cancers which develop in BRCA1-mutant carriers will have TNBC and the molecular mechanism facilitating tumor development is unclear. Our earlier work suggested Ubc9 to play a critical role in BRCA1 loss mediated TNBC cell migration and metastasis. Collagen is one of the major components of the stromal extracellular matrix (ECM) network that influences tissue density. Its re-organization act as a scaffold aiding cancer cells to migrate causing metastasis. Ubc9 is known to increase the production of collagen, a key component of fibroglandular breast tissue, as well as tumorigenesis. Our work is based on the hypothesis that loss of BRCA1 in women with high breast density causes abnormal Ubc9 levels which upregulates collagen, fibronectin and inhibits SIRT1, β-catenin expression facilitating TNBC. We tested this hypothesis by studying the expression of total collagen, fibronectin, Ubc9, SIRT1, β-catenin in BRCA1 mutant TNBC cells and tumor sample derived from patient with dense breasts using immunofluorescence, immunohistochemistry, and collagen assay. Our results suggest for the first time that mutation or loss of BRCA1 function in women with fibrocystic breasts can lead to over expression of Ubc9, induction of collagen and; fibronectin, inhibition of SIRT1 and nuclear accumulation of β-catenin which could contribute to TNBC development. This network will aid not only in the identification of potential mechanism-based biomarkers that could detect disease early, but also enforce preventive measures that could reduce the risk for TNBC in women with high MD thus reducing the mortality associated with these cancers to achieve health equity.

## Introduction

Breast cancers are represented by a heterogeneous group of tumors. They are characterized by a wide spectrum of clinical, pathological and molecular features as well as by their responses to therapy [1]. Among women in the US, breast cancer is the most common cancer diagnosis (excluding skin cancer), and it is the leading cause of cancer death second to lung cancer [2]. According to the American Cancer Society, an estimated 268,600 new cases of female breast cancer will be diagnosed in women in the U.S in 2018 and an estimated 41,760 will die from it [2]. Recently, more attention has been devoted to the classification of primary breast cancer into molecular subtypes using markers like estrogen receptor (ER), progesterone receptor (PR) and human epidermal growth factor receptor (HER) [3,4]. Luminal subtypes make up the hormone receptor–expressing tumors and generally carry a favorable prognosis. Human epidermal growth factor receptor 2 (HER2) subtypes refer to predominantly hormone receptor–negative tumors with a specific gene expression pattern [4–7]. TNBC - defined by the lack of ER, PR and HER-2 receptors, - is a highly aggressive subtype of breast cancer and accounts for 15–20% of all breast cancer cases [5,8–11]. Interestingly, about 70% of TNBC is associated with basal like subtype and 76% of basal like cancers are triple negative. Histologically, TNBC is high grade with a high mitotic index and can be either invasive ductal carcinoma or metaplastic carcinoma [3,12]. TNBC is a pressing area of research for both researchers and clinicians alike because (1) TNBC is a poor prognostic factor for disease-free and overall survival, (2) no targeted therapy is readily available for TNBC, (3) there is a clustering of TNBC cases specifically in premenopausal women and in women of African descent, and (4) the overlap of BRCA1-associated breast cancers with the TNBC phenotype is significant [1,3,10,13–15].

Although the incidence rates of breast cancer between Non-Hispanic white women and AA women are getting closer, the mortality rates in AA women are higher [16]. TNBC is a heterogeneous disease group divided into six different subtypes based on gene expression analysis/profiles: BL1, BL2, IM, M, MSL, and LAR [17]. The classification of TNBC into molecular subtypes is essential for developing personalized treatments as each subtype responds uniquely to specific treatments. For example, BL1 and BL2 are both of the “basal-like” type and initially are sensitive to platinum drugs, such as cisplatin. M and MSL are both of the “mesenchymal” type and are initially sensitive to kinase inhibitors, such as dasatinib. IM is of the “immunomodulatory” type, and LAR is of the “luminal” type and is initially sensitive to non-steroidal androgen receptor hormone therapy, such as bicalutamide [17].

Although these are a few of the current treatments used, there is still no targeted therapy for TNBC as there exists for other types of breast cancers. In addition to the lack of effective treatment for it, the basal-like subtype is the most aggressive and, considering the statistics above, is unsurprisingly most prevalent in AA women [18,19]. Notably, tumors with BRCA1 mutation are also of the basal-like subtype [20].

Women who are germline BRCA1 mutation carriers have a 15% increased risk of developing breast cancer and, if diagnosed, a 40–60% risk of a TNBC diagnosis. In fact, over 75% of breast tumors found in women with a BRCA1 mutation are of the TNBC subtype [21]. As with breast cancer mortality and prevalence of the basal-like subtype, the frequency of BRCA1 mutations is higher in AA women. A recent study, in which 289 self-identified AA patients, all of whom had been diagnosed with primary invasive breast cancer, were evaluated for a variety of germline mutations, including BRCA1 mutations, found that 80% of these patients carried mutations in their BRCA1 and BRCA2 genes. Other studies have also shown that there are higher frequencies of BRCA1 mutations in breast cancer patients of African descent, especially descendants with an origin of ancestry from Nigeria and the Bahamas [18,22].

SUMOylation has been shown to regulate transcription factor activities that are involved in several cellular signaling pathways, such as: BRCA1, cell cycle, and steroid hormones in breast cancer pathogenesis [23–26]. We have shown that wild type BRCA1 proteins, unlike the disease-associated mutant BRCA1 proteins, bind the sole SUMO E2-conjugating enzyme Ubc9 and function as a tumor suppressor [25]. BRCA1 is a multifunctional protein that activates ER-a transcriptional activation and tumor suppression [21]. BRCA1 mutation, deficiency or abnormal subcellular localization causes elevated Ubc9 levels and ER-α repression leading to TNBC [24,25]. Using patient-derived cell lines with known BRCA1 mutations, we reported the *in-vivo* association of BRCA1 and Ubc9 in normal mammary epithelial cells, but not in BRCA1 mutant TNBC cells. We observed an increased expression of Ubc9 in BRCA1 mutant TNBC cells while knockdown of Ubc9 expression in these cells decreased their proliferation and migration [27]. For the first time, we were able to show that high Ubc9 expression due to BRCA1 mutation may trigger an early growth and transformation advantage to normal breast and ovarian epithelial cells resulting in aggressive cancers [27]. In addition to TNBC, it has also been reported that Ubc9 is either upregulated or over-expressed in other tumor cells as well, such as: ovarian, lung, head, neck, and melanoma etc., [28].

Breast tissue architecture has a central role in breast biology and function along with other tissue biophysical parameters like X-ray density and mechanical stiffness. Mammography is the most common imaging technique used for the screening of breast cancer and it can help in differentiating certain biophysical markers of human breast, such as mammographic density (MD) [29,30]. The classification of breast density can be identified as “low” density or “high” density. MD is defined by the ratio of fibroglandular breast tissue (epithelial cells, fibroblasts, and connective tissue) to the amount of adipose tissue in the breast. The higher the percentage of fibroglandular tissue, the denser the breast [31,32]. Collagen is produced by fibroblasts and is a key component in fibroglandular breast tissue. Dense breast tissue gives a light appearance on the mammogram and is composed of stromal and epithelial cells, whereas non-dense breast appears darker and is composed of adipose tissue [13].

Recently, there have been studies showing a positive correlation between mammographic breast density and risk for the development of breast cancer. This association has caused breast density to emerge as a critical phenotypic marker of increased breast cancer risk, resulting in mammographic breast density to be one of the strongest known risk factors for breast cancer. In fact, the only other two risk factors that are stronger are BRCA1 mutations and age [33]. Women with over 75% dense tissue have four to six times the risk of breast cancer due to the increased fibroglandular tissue content, compared to those with very little to no dense tissue [12,21,31,32]. Furthermore, high mammographic breast density has also been positively associated with breast tumor characteristics that are predictive of worse prognosis including larger tumor size, positive lymph nodes, and advanced stage. It seems logical then that tumors in high MD breasts may also progress more rapidly [34].

Recently, it was found that breast density has a strong association with ER-negative diseases, such as those common in the TNBC subtypes. Additionally, it was predicted that tumors in dense breast have increased interaction with stromal and epithelial cells, resulting in more aggressive tumors [35]. This theory suggests a mechanism by which fibroglandular breast tissue may play a role in tumorigenesis. To that effect, it has also been shown that a positive correlation exists between TNBC and *COL4A2* in a study conducted by Jing Song et al. [36]. It was found that collagen type IV alpha 2 (*COL4A2*) was highly expressed in TNBC. Additionally, when cells were treated with collagen siRNA1 and siRNA2, the number of cells undergoing early apoptosis increased significantly. When the collagen was inhibited, a higher percentage of cells were found in the G2 phase while a lower percentage was found in the S phase [36]. Ubc9 plays a critical role in the production of collagen, a key component of fibroglandular breast tissue, as well as tumorigenesis. In a rheumatoid arthritis (RA) study conducted by Li et al. [37], the role of Ubc9 in the progression of RA was shown using a collagen-induced arthritis (CIA) model. The expression levels of Ubc9 were first observed in the joints of the CIA model and compared to the levels of Ubc9 in the normal joints model. The study found that the CIA joints had a significantly higher Ubc9 expression. Once Ubc9 was inhibited by treatment with Ubc9 siRNA, there was a significant reduction in the arthritis score and joint destruction [37]. This study provides evidence that Ubc9 plays a critical role in collagen production. Recently, De Filipis et al. have shown high MD to be negatively associated with CD36, an integral membrane protein that regulates many cellular processes such as differentiation of adipocytes [38]. In a separate study of brown adipose fat in Type 2 Diabetes patients, Ubc9 knockdown increased the expression of CD36, suggesting a mechanism by which Ubc9 down regulates brown adipose tissue deposition [39]. They further showed that reducing the expression of CD36 resulted in a decrease in the amount of fat cells and an increase in the amount of collagen in the ECM, both of which would lead to a high MD.

Fibronectin (FN) is a glycoprotein of the extracellular matrix that plays a major role in cell adhesion, migration, and oncogenic transformation [40]. SIRT1, a nicotinamide adenine dinucleotide (NAD)-dependent histone-deacetylase, is linked to longevity, metabolism, stress response, genomic stability and energy homeostasis [41]. Our recent work suggests for the first time a novel molecular mechanism by which BRCA1 by tethering Ubc9 induces SIRT1, caveolin-1, and ER-a expression inhibiting TNBC metastasis to the lung [42]. In fact, SIRT1 expression was found to be significantly reduced in TNBC subtype [43] which correlates with our observation. Wild-type BRCA1, but not the mutated BRCA1 regulates the expression of the nuclear, active non-phosphorylated form of β-catenin *in vitro* [44]. Sumoylation of β-catenin was shown to be involved in the deregulated proliferation of myeloma cells [45]. Our data suggests for the first time how BRCA1 dysfunction results in deregulated expression of Ubc9, induction of collagen, fibronectin, inhibition of SIRT1 and nuclear active form of β-catenin contributing to EMT in women with high MD leading to TNBC.

## Materials and Methods

### Cell culture

HCC1937; and HCC1937 BRCA1 cells were obtained from American Type Culture Collection (Rockville, MD, USA), CAL51 cells were obtained from CD Biosciences Inc. and cultivated as described previously [25]. Stable cell line HCC1937 BRCA1a, CAL51 BRCA1a Mut#1 has been described previously [42]. CAL51 cells were grown in DMEM medium with 10% FBS, 0.6 μg/ml Insulin, 5 × 10^3^ μg/ml transferrin and 1% PS. HCC1937 cells were grown in RPMI 1640 medium with 10% FBS and 1% PS.

### Expression plasmids

The BRCA1/1a expression plasmids BRCA1a, BRCA1a Mut#1 (K109R), BRCA1 C7-C51(1–182 amino acids (aa), BRCA1 C7-C51 Mut#1(K109R) were cloned as described previously [25].

### Antibodies and reagents

The antibodies used in this study were primary polyclonal Rabbit anti-Collagen antibody (Santa Cruz biotechnology), primary polyclonal Rabbit anti-Fibronectin antibody (Santa Cruz Biotechnology), primary polyclonal Rabbit anti-β-Catenin antibody (Santa Cruz Biotechnology), primary polyclonal Goat anti-Ubc9 N-15 antibodies (Santa Cruz Biotechnology).

### Immunofluorescence analysis

HCC1937, HCC1937 BRCA1, and CAL51 cells were cultured alone or transfected with pCDNA3, BRCA1a, BRCA1a Mut#1, for 24 hours in six-well plates onto glass cover slips overnight. The cells were washed and fixed in icy methanol for 5 minutes, and blocked using 10% BSA for 60 minutes, followed by primary polyclonal Rabbit anti-collagen antibody 1:250, primary polyclonal anti-fibronectin and β-catenin antibody (Santa Cruz), 1:500 diluted in 1.5% BSA made in PBS at 25°C for 1 hour and Alexa 488 goat anti-Rabbit/Alexa 568 goat anti-mouse (Molecular Probes) diluted in 1.5% BSA/PBS for 50 minutes and stained (Hoechst 33258, Pentahydrate, Life technologies). The cover slips were mounted with Vectashield mounting medium for fluorescence (H-1000 from Vector). The stained cells were examined by LSM 700 Confocal Microscope, equipped with 63X oil Ph immersion objectives. Composite filter cubes were used for the 488–405 as described previously [25].

### Collagen assay

HCC1937 and HCC1937 pCDNA3 cells were homogenized in distilled H_2_O and an equal amount of 12M HCl was added so the samples were hydrolyzed at 120°C for 3 hrs. Samples were clarified with active charcoal be evaporated to dryness under a vacuum in a 96-well plate. Samples were measured at 560 nm after 90 minutes incubation at 60°C with Chloramine T reagent and compared to collagen standard.

### Patient selection and data collection

The surgical pathology files at Grady Memorial Hospital and Emory University Hospital were searched for breast carcinomas that did not immunohistochemically (IHC) stain for ER, PR, and HER2 neu. When HER2 was equivocally expressed on IHC, the amplification status was further studied by fluorescence *in situ* hybridization (FISH) for HER2. These comprised the TNBC’s. In addition to clinical pathology data and genetic mutation, whole slides were examined for the presence of tumor and for the appearance of benign tissue adjacent to the tumor. Cases were selected based on whether there was enough tumor available for staining. Individual patient data was de-identified, and there was no patient contact. This study did not pose any risk to patients and was approved by the Institutional Review Board.

### Hormone receptor and HER studies

At the time of diagnosis, the predictive and prognostic breast carcinoma markers ER (clone 6F11 Novocastra), PR (clone 16 Novocastra) and HER2 neu (clone CB11 Novocastra) were performed on formalin fixed, paraffin embedded (FFPE) tissue and evaluated following College of American Pathologists (CAP) guidelines. When HER2 was equivocally expressed on IHC, the amplification status was further studied by fluorescence *in situ* hybridization (FISH) for HER2. The scoring of the IHC results was semi quantitative, using 0–3 for intensity and a percentage of tumor cells staining, following the current American Society of Clinical Oncology and the College of American Pathologists guidelines [46]. In tumors with a HER grade of IHC 2+, FISH was used to further determine the overexpression of HER.

### Tissue Microarrays (TMAs)

All microscopic slides from TNBC were reviewed, and the most representative tumor block was selected as the donor block for the TMA. Two 1-mm cores from each tumor were included in the TMA recipient block.

### Immunohistochemistry

Slides (4 microns) were cut from the TMAs and were evaluated for Ubc9 IHC staining. Goat monoclonal antibody (ab21193, Abcam) (1:200 dilution) was used as primary antibody. Goat IgG was added as negative control. The tissue was pretreated at 65°C for 2 hours, followed by deparaffinization using standard procedures. Antigen retrieval was carried out in antigen retrieval solution (10 mmol/l Tris, 1 mmol/l EDTA, pH 9.0) before sheep serum was added. Then slides were incubated with Ubc9 antibody for more than 16 hours at 4°C, overnight. After 3 washes with PBST and further incubation with horseradish peroxidase (HRP)-conjugated secondary antibody for 1 hour at 37°C, positive signals were detected with the chromogen 3,3’-diamiobenzidine (DAB). Positive expression of Ubc9 was mainly detected in the cytoplasm and/or nucleus. For the staining, Ubc9 clone Ep298Y, Novus biological was used in dilution of 1:100. For Ubc9 antigen retrieval was performed by microwaving slides at 800 W for 10 minutes followed by 560 W for 10 minutes in citrate buffer. (1M sodium citrate at pH of 6.0) and cooling in running water immediately. The primary antibody for the biomarker was incubated for 60 minutes at room temperature. Diaminobenzidine tetra hydrochloride (DAB) solution was incubated for 10 minutes after which copper-sulphate solution (0.5% copper sulfate in 0.8% sodium chloride) was applied to the slides and incubated for 10 minutes and counterstained with hematoxylin for 2–3 minutes, followed by rinsing in tap water. Slides were de-hydrated by immersing in three alcohol baths for 10 seconds and cleared in two xylene baths followed by application of cover slip. Negative and positive controls were performed by omitting the primary antibody and including control tissue as specified by the manufacturer, respectively. The scoring of the IHC results was semi quantitative, using the percentage of positive cells to produce a final score in the range 0 to100. The cases were scored without knowledge of the clinical pathology parameters or patient outcome. For Ubc9 the TMA were scored twice. The staining of the two core samples from each tumor was evaluated separately. The average of the individual cores was used for the final score.

## Results and Discussion

### BRCA1a inhibits the expression of collagen in BRCA1-deficient HCC1937 and CAL51 TNBC cells

**Figure 1.**
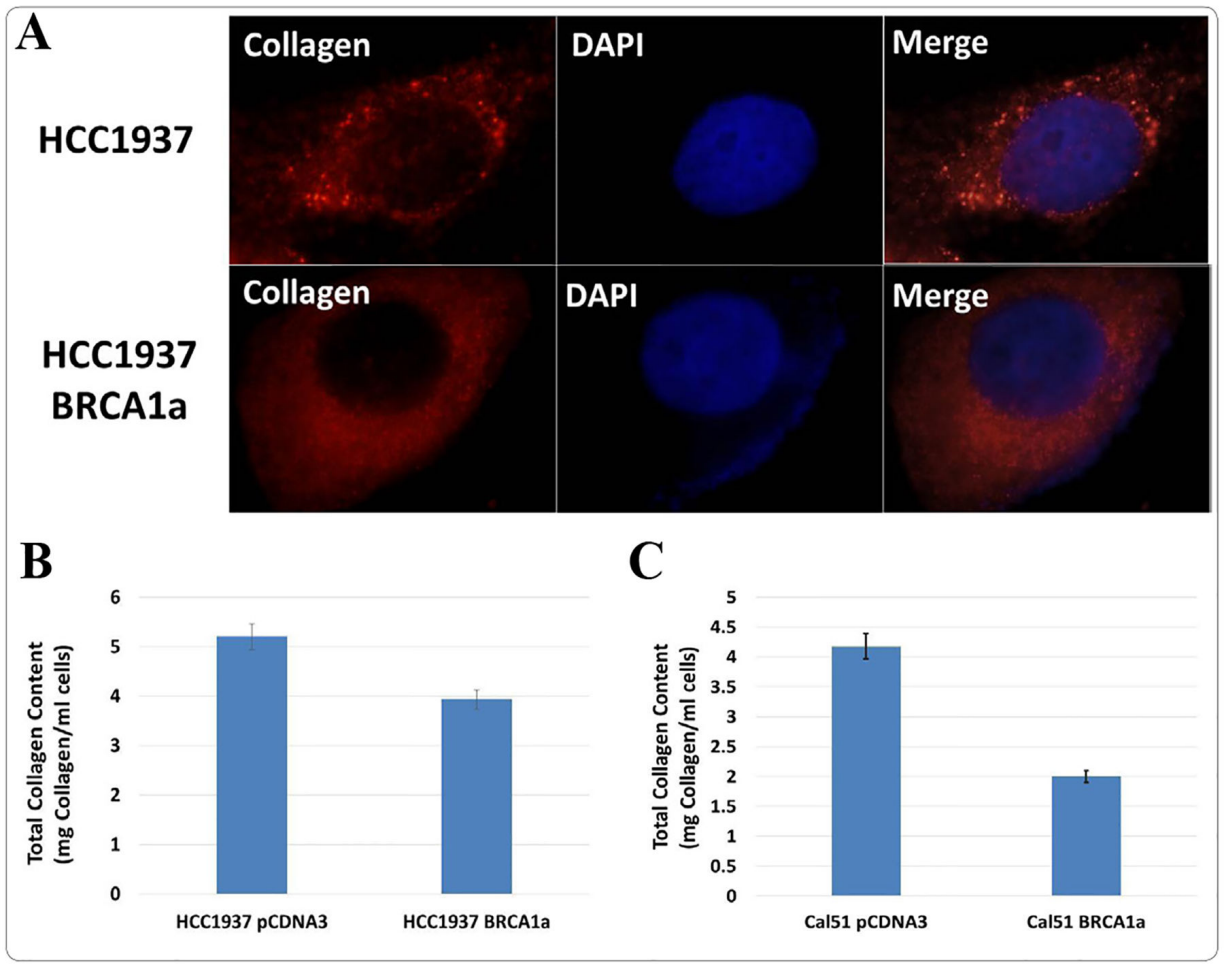
Collagen content is higher in BRCA1 defective HCC1937 and CAL51 TNBC cells compared to HCC1937-BRCA1a and Cal51-BRCA1a cells. **A.** HCC1937 and HCC1937-BRCA1a stable cells were seeded into six-well plates. The nuclei were visualized with DNA staining Hoechst 33258. Cells were fixed in icy methanol and probed with collagen (Santa Cruz, 1/250) followed by Alexa Fluor 568 labeled secondary antibody (Invitrogen, 1/200) staining as described previously [25]. The images were taken using a fluorescent microscope (100X, oil Olympus). **B.** Total collagen content is higher in HCC1937 cells compared to HCC1937 BRCA1a cells **C.** Total collagen content is higher in CAL51 cells compared to CAL51 BRCA1a cells as estimated using collagen assay kit (Bio Vision). HCC1937 pCDNA3, HCC1937 BRCA1a, CAL51 pCDNA3 and CAL51 BRCA1a cell lysates were homogenized in double distilled H_2_O and hydrolyzed with 12 M HCl for 3 hours at 120°C. Precipitates were removed by centrifugation (10000 × g, 3 min). Thirty micro liters of the hydrolyzed samples were assayed using a collagen assay kit according to the manufacturer’s protocols. Collagen content (mg collagen/g cells): HCC1937 pCDNA, 3 5.2 ± 0.3; HCC1937 WT, 3.9 ± 0.2; CAL51 pCDNA, 4.18 ± 0.3; CAL51 BRCA1a, 2 ± 0.1 respectively. Statistical analysis for collagen assay was done as described by us previously [27].

**Figure 2.**
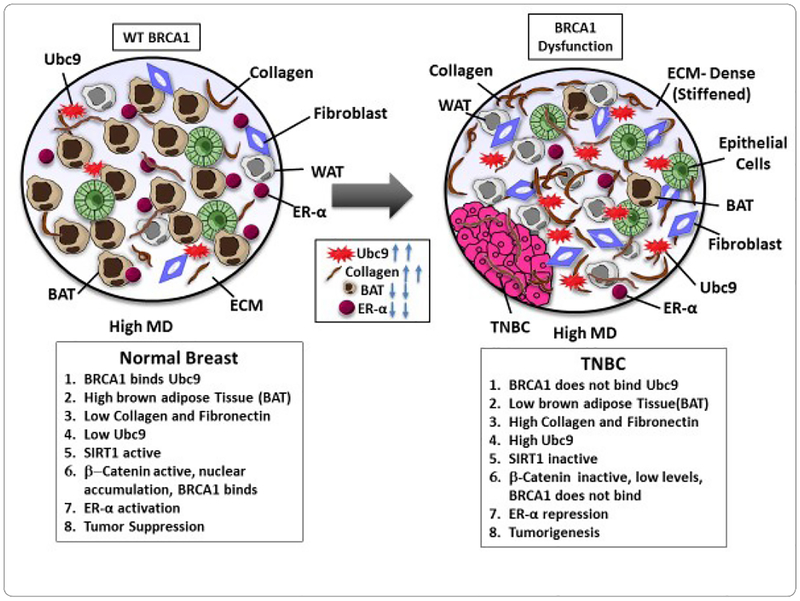
Working hypothesis showing how BRCA1 mutation/dysfunction in patients with dense breasts can promote TNBC development.

To study whether BRCA1a inhibits collagen expression in BRCA1-mutant HCC1937 and CAL51 TNBC cells, we have studied the expression of collagen in these cells by using immunofluorescence and collagen assay kits. Our results show higher levels of expression of collagen in HCC1937 cells compared to HCC1937 cells stably transfected with BRCA1a by immunofluorescence analysis using collagen antibodies (Figure 1A). Similarly, collagen content was found to be higher in HCC1937 pCDNA3 and CAL51 pCDNA3 cells compared to BRCA1a transfected HCC1937 and CAL51 cells using the total collagen assay kit (Figures 1B and 1C). These results support our hypothesis that loss of binding of mutant/defective BRCA1 to Ubc9 in TNBC could cause increased collagen expression which may contribute to the pathogenesis of TNBC (Figure 2).

### Ubc9 is expressed at elevated levels in BRCA1 mutant TNBC tumor tissue obtained from an AA woman with high breast density

**Figure 3.**
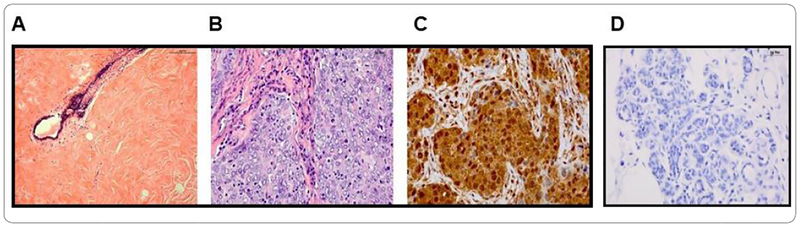
Ubc9 is expressed at elevated levels in BRCA1 mutant TNBC tissue from a patient with dense breasts. **A.** This microphotograph depicts dense breast tissue adjacent to the tumor depicted in figure 2B & 2C. The patient had BRCA1 mutation (c2806_2809delGATA pathogenic). Benign breast ducts are surrounded by dense fibrous stroma (HE 10X). **B.** This photomicrograph represents a triple negative breast carcinoma from a patient with BRCA1 mutation. The high magnification better illustrates the high-grade tumor composed of sheets of malignant cells with ill-defined cell borders and syncytial-like appearance. There is a fair amount of cytoplasm. Most of the nuclei show chromatin vesiculization with prominent nucleoli. Mitotic figures are conspicuous and frequent. (HE 40X). **C.** Immunostaining for Ubc9 is illustrated in this microphotograph. The tumor cells show strong nuclear and cytoplasmic staining (40X). **D.** Immunostaining for Ubc9 using a matched benign tissue sample.

Our group has previously found BRCA1 proteins, unlike the pathogenic mutant BRCA1/1a proteins to bind Ubc9 [25] and function as a tumor suppressor in TNBC cells [25,27]. Furthermore, positive Ubc9 expression was found to correlate with poor clinical outcome in Nigerian women with TNBC [23]. To study whether Ubc9 is expressed at elevated levels in TNBC tumor tissue obtained from an AA woman with high MD and BRCA1 mutation, we studied Ubc9 expression in this sample by IHC staining using Ubc9 antibody. We observed increased expression of Ubc9 in TNBC tissues obtained from AA women with high MD (Figure 3) compared to match benign tissue using IHC analysis suggesting a potential role for Ubc9 in triggering TNBC development. A finding once again reinforces a role for Ubc9 in the development of BRCA1-linked TNBC.

### BRCA1 unlike the Ubc9 binding BRCA1 mutant inhibits fibronectin protein expression in TNBC cells

**Figure 4.**
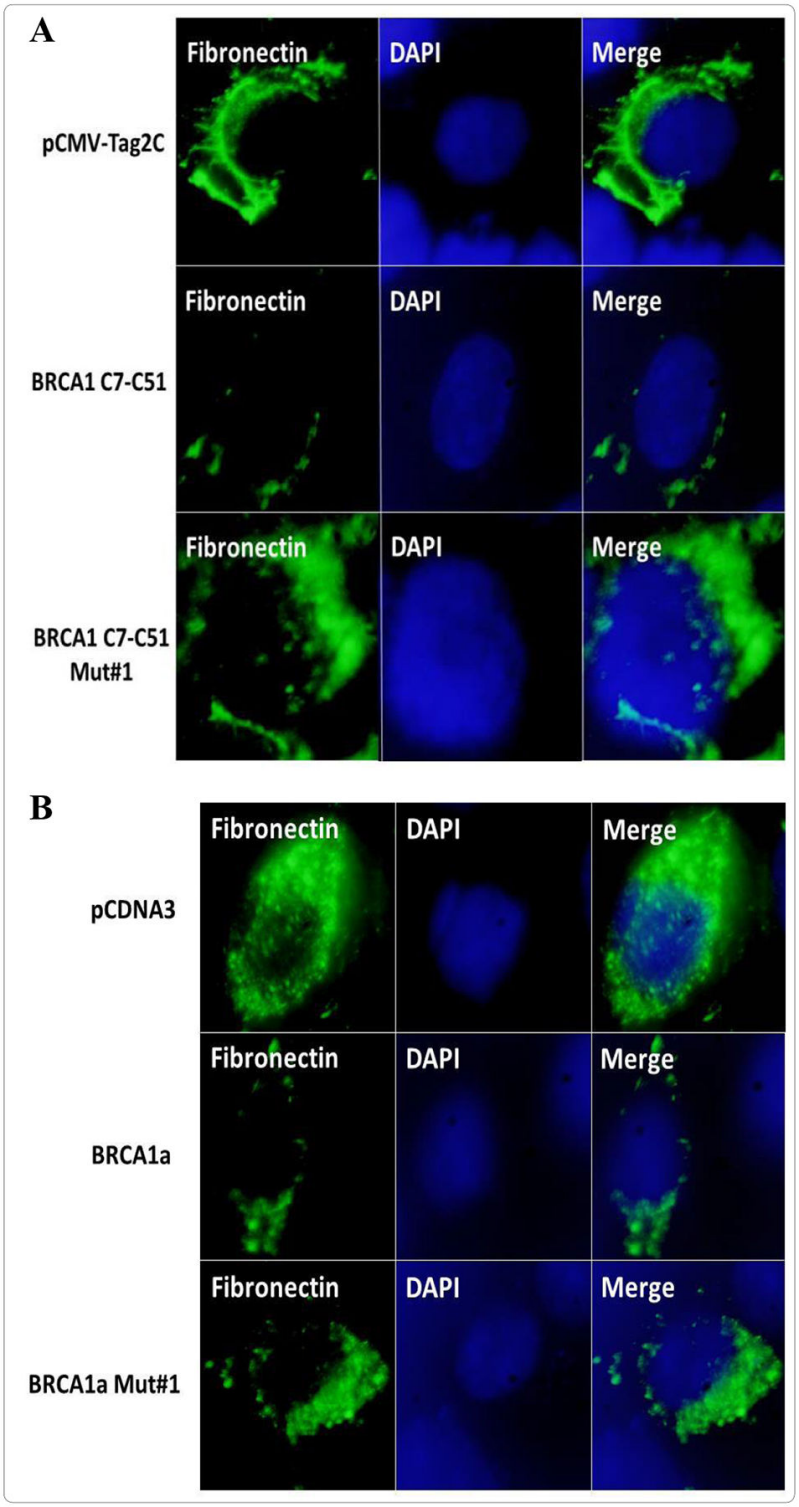
BRCA1 domain (1–182aa) unlike BRCA1 (1–182aa)-K109R is sufficient to inhibit fibronectin levels in TNBC cells. **A.** BRCA1a but not BRCA1a Mutant #1 (K109R) inhibits fibronectin expression in the CAL51 TNBC cell line as seen by immunofluorescence analysis. **B.** BRCA1 C7-C51 (1–182 aa) and not BRCA1 C7-C51 Mutant #1 (K109R) is enough to inhibit fibronectin expression in CAL51 cells as seen using immunofluorescence analysis. The fibronectin protein was detected by immunostaining using anti-fibronectin antibody. DAPI fluorescent staining is shown in parallel. CAL51 cells were transfected with pCMV-Tag2C vector alone or BRCA1 C7-C51 or BRCA1 C7-C51 Mutant #1 and seeded into six-well plates. The nuclei were visualized with DNA staining DAPI. Cells were fixed in icy methanol and probed with fibronectin antibody (Santa Cruz, 1/500) followed by Alexa Fluor 488 labeled secondary antibody (Invitrogen, 1/200) staining as described previously [25]. The images were taken using fluorescent microscope (100X, oil Olympus).

Fibronectin is an adhesive glycoprotein of the extracellular matrix that is known is known to play a role in cell adhesion, migration in various human cancers and is expressed at elevated levels in TNBC [40]. To investigate whether wild type BRCA1a downregulates the expression of fibronectin in TNBC cells, we studied the expression of fibronectin in CAL51 TNBC cells in the absence and presence of wild type BRCA1a and BRCA1a Mut#1(K109R) by immunofluorescence analysis using fibronectin antibodies. Our results show inhibition of fibronectin levels in BRCA1a unlike BRCA1a Mut#1 (Figure 4A). Similarly, we repeated the experiments using a carboxy terminal truncated BRCA1 C7-C51(aa1–182) and BRCA1 C7-C51 Mut#1 (K109R). We observed inhibition of fibronectin levels in BRCA1 C7-C51 unlike BRCA1 Mut#1 in CAL51 TNBC cells (Figure 4B). Since the BRCA1 Mut#1 does not bind to Ubc9 it suggests that binding of BRCA1 to Ubc9 may be required for downregulation of fibronectin levels in TNBC cells. It also suggests that the NH2 terminal BRCA1–182 aa is enough for inhibition of fibronectin levels in TNBC cells.

### BRCA1a increases the nuclear β-catenin protein expression in TNBC cells

**Figure 5.**
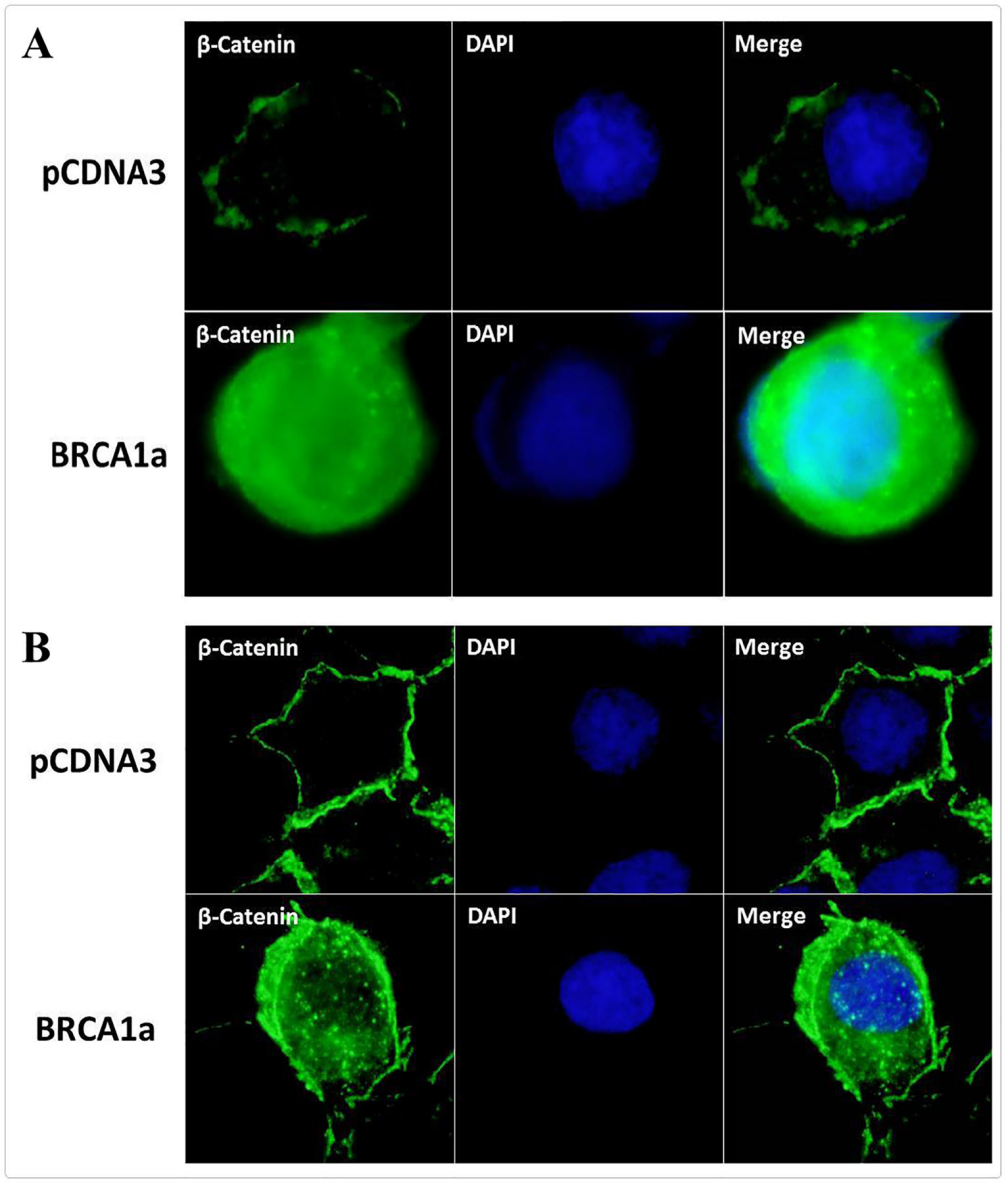
BRCA1a increases β-Catenin protein expression in TNBC cells. **A.** HCC1937 pCDNA3, HCC1937 BRCA1a. **B.** CAL51 pCDNA3 and CAL51 BRCA1a cells were seeded overnight and subjected to immunofluorescence analysis using anti-β-catenin antibody. DAPI staining is shown in parallel. The active non-phosphorylated form of β-catenin is in the nucleus. Cells were washed and fixed in icy methanol for 5 minutes, and blocked using 10% BSA for 60 minutes, followed by primary Polyclonal anti-β-catenin antibody (Santa Cruz, 1/500) 1:150 diluted in 1.5% BSA/PBS at 25°C for 1 hour and Alexa 488 goat anti-Mouse antibody (Molecular Probes) 1:100 diluted in 1.5% BSA/PBS for 50 minutes, in combination with DAPI staining. The cover slips were mounted with Vectashield mounting medium (Vector) for fluorescence. The stained cells were examined by LSM 700 Confocal Microscope, equipped with63X oil Ph immersion objectives.

Both β-catenin and BRCA1 proteins were shown to be required for normal mammary gland development. BRCA1, but not mutated BRCA1, was shown to interact and increase the levels of non-phosphorylated and active nuclear form of β-catenin in TNBC cells [44]. This group provided the first evidence to suggest that loss of BRCA1 in TNBC cells leads to impaired expression of nuclear form of β-catenin which can lead to breast cancer [44]. To examine whether BRCA1a, the isoform of BRCA1 induces β-catenin expression in TNBC, we studied the expression of β-catenin in HCC1937 pCDNA3, HCC1937 BRCA1a, CAL51pCDNA3, and CAL51 BRCA1a stable cells lines using β-catenin antibodies by immunofluorescence analysis (Figures 5A and 5B). Our results show nuclear β-catenin to be induced both in HCC1937 BRCA1a and CAL51 BRCA1a cells compared to vector transfected HCC1937 and CAL51 TNBC cell lines. The results suggest BRCA1a to be like BRCA1 in regulating β-catenin protein expression in TNBC cells [45].

## Conclusions

TNBC is the most invasive and aggressive breast cancer that has a high mortality rate and disproportionately affects young AA women who have a much higher frequency of BRCA1 mutations [18,19,22]. There have been studies showing a positive correlation between high MD and the risk for the development of TNBC but the molecular pathway facilitating tumor development is not understood [12,13,31,35]. Collagen one of the major components of the stromal ECM network influences tissue density [36,38], and its reorganization acts as a scaffold aiding cancer cells to migrate causing metastasis [36]. Our previous work suggested Ubc9 to play a critical role in BRCA1 loss mediated TNBC metastasis [42]. Ubc9 is shown to increase the production of collagen, a key component of the fibroglandular breast tissue, as well as, tumorigenesis [37]. Our results suggest for the first time that mutations or loss of BRCA1 function in women with high MD can lead to overexpression of Ubc9, induction of collagen, fibronectin, and inhibition of SIRT1 and; cytoplasmic localization of β-catenin which could rapidly progress to TNBC development. This study highlights the need for not only frequent breast screening and, use of digital mammography but also screening for potential mechanism-based biomarkers (like Ubc9, SIRT-1, β-catenin, etc.) that can predict TNBC early in women with high mammography density and BRCA1 mutation thus reducing the mortality associated with these aggressive cancers leading to health equity.

## Abbreviations:

TNBC: Triple Negative Breast Cancers
ER: Estrogen Receptor
AA: African American
MD: Mammographic density
BAT: Brown Adipose Tissue
WAT: White Adipose Tissue
EMT: Epithelial Mesenchymal Transition
ECM: Extracellular Matrix
SIRT1: Sirtuin1

## References

[1] Rakha EA, Elsheikh SE, Aleskandarany MA, Triple-negative breast cancer: distinguishing between basal and nonbasal subtypes. Clin Cancer Res. 2009; 15(7): 2302–2310. doi: 10.1158/1078-0432.CCR-08-2132 19318481

[2] Siegel RL, Miller KD, Jemal A. Cancer statistics, 2018. CA Cancer J Clin. 2018; 68(1): 7–30. doi: 10.3322/caac.21442 29313949

[3] Kreike B, van Kouwenhove M, Horlings H, Gene expression profiling and histopathological characterization of triple-negative/basal-like breast carcinomas. Breast Cancer Res. 2007; 9(5): R65. doi: 10.1186/bcr1771 17910759 PMC2242660

[4] Rody A, Karn T, Liedtke C, A clinically relevant gene signature in triple negative and basal-like breast cancer. Breast Cancer Res. 2011; 13(5): R97. doi: 10.1186/bcr3035 21978456 PMC3262210

[5] Dent R, Trudeau M, Pritchard KI, Triple-negative breast cancer: clinical features and patterns of recurrence. Clin Cancer Res. 2007; 13: 4429–4434. doi: 10.1158/1078-0432.CCR-06-3045 17671126

[6] Peddi PF, Ellis MJ, Ma C. Molecular Basis of Triple Negative Breast Cancer and Implications for Therapy. Int J Breast Cancer. 2012; 2012: 217185. doi: 10.1155/2012/217185 22295242 PMC3262606

[7] Haffty BG, Yang Q, Reiss M, Locoregional relapse and distant metastasis in conservatively managed triple negative early-stage breast cancer. J Clin Oncol. 2006; 24(36): 5652–5657. doi: 10.1200/JCO.2006.06.5664 17116942

[8] Adamo B, Anders CK. Stratifying triple-negative breast cancer: which definition(s) to use? Breast Cancer Res. 2011; 13(2): 105. doi: 10.1186/bcr2852 21457488 PMC3219193

[9] Boisserie-Lacroix M, Macgrogan G, Debled M, Triple-negative breast cancers: associations between imaging and pathological findings for triple-negative tumors compared with hormone receptor-positive/human epidermal growth factor receptor-2-negative breast cancers. Oncologist. 2013; 18(7): 802–811. doi: 10.1634/theoncologist.2013-0380 23821326 PMC3720633

[10] Ouyang M, Li Y, Ye S, MicroRNA profiling implies new markers of chemoresistance of triple-negative breast cancer. PLoS One. 2014; 9(5): e96228. doi: 10.1371/journal.pone.0096228 24788655 PMC4008525

[11] Teng YH, Tan WJ, Thike AA, Mutations in the epidermal growth factor receptor (EGFR) gene in triple negative breast cancer: possible implications for targeted therapy. Breast Cancer Res. 2011; 13(2): R35. doi: 10.1186/bcr2857 21457545 PMC3219198

[12] Chen JH, Agrawal G, Feig B, Triple-negative breast cancer: MRI features in 29 patients. Ann Oncol. 2007; 18(12): 2042–2043. doi: 10.1093/annonc/mdm504 18029970

[13] Stead LA, Lash TL, Sobieraj JE, Triple-negative breast cancers are increased in black women regardless of age or body mass index. Breast Cancer Res. 2009; 11(2): R18. doi: 10.1186/bcr2242 19320967 PMC2688946

[14] Ismail-Khan R, Bui MM. A review of triple-negative breast cancer. Cancer Control. 2010; 17(3): 173–176. doi: 10.1177/107327481001700305 20664514

[15] Banerjee S, Reis-Filho JS, Ashley S, Basal-like breast carcinomas: clinical outcome and response to chemotherapy. J Clin Pathol. 2006; 59(7): 729–735. doi: 10.1136/jcp.2005.033043 16556664 PMC1860434

[16] American Cancer Society. Breast Cancer Facts & Figures 2017–2018. 2017.

[17] Lehmann BD, Bauer JA, Chen X, Identification of human triple-negative breast cancer subtypes and preclinical models for selection of targeted therapies. J Clin Invest. 2011; 121(7): 2750–2767. doi: 10.1172/JCI45014 21633166 PMC3127435

[18] Churpek JE, Walsh T, Zheng Y, Inherited predisposition to breast cancer among African American women. Breast Cancer Res Treat. 2015; 149(1): 31–39. doi: 10.1007/s10549-014-3195-0 25428789 PMC4298662

[19] Dietze EC, Sistrunk C, Miranda-Carboni G, O’Regan R, Seewaldt VL. Triple-negative breast cancer in African American women: disparities versus biology. Nat Rev Cancer. 2015; 15(4): 248–254. doi: 10.1038/nrc3896 25673085 PMC5470637

[20] Sorlie T, Tibshirani R, Parker J, Repeated observation of breast tumor subtypes in independent gene expression data sets. Proc Natl Acad Sci U S A. 2003; 100(14): 8418–8423. doi: 10.1073/pnas.0932692100 12829800 PMC166244

[21] Aysola K, Desai A, Welch C, Triple Negative breast cancer-an overview. Hereditary Genet. 2013. doi: 10.4172/2161-1041.S2-001 PMC4181680 25285241

[22] Pal T, Bonner D, Cragun D, A high frequency of BRCA mutations in young black women with breast cancer residing in Florida. Cancer. 2015; 121(23): 4173–4180. doi: 10.1002/cncr.29645 26287763 PMC4666784

[23] Agboola AO, Musa AA, Ayoade BA, Clinicopathological and molecular significance of Sumolyation marker (ubiquitin conjugating enzyme 9 (UBC9)) expression in breast cancer of black women. Pathol Res Pract. 2014; 210(1): 10–17. doi: 10.1016/j.prp.2013.09.011 24176171

[24] Xu J, Watkins T, Reddy A, Reddy ES, Rao VN. A novel mechanism whereby BRCA1/1a/1b fine tunes the dynamic complex interplay between SUMO-dependent/independent activities of Ubc9 on E2-induced ERalpha activation/repression and degradation in breast cancer cells. Int J Oncol. 2009; 34(4): 939–949. doi: 10.3892/ijo_00000220 19287951 PMC7983531

[25] Qin Y, Xu J, Aysola K, Ubc9 mediates nuclear localization and growth suppression of BRCA1 and BRCA1a proteins. J Cell Physiol. 2011; 226(12): 3355–3367. doi: 10.1002/jcp.22695 21344391 PMC3329759

[26] Moschos SJ, Jukic DM, Athanassiou C, Expression analysis of Ubc9, the single small ubiquitin-like modifier (SUMO) E2 conjugating enzyme, in normal and malignant tissues. Hum Pathol. 2010; 41(9): 1286–1298. doi: 10.1016/j.humpath.2010.02.007 20561671

[27] Xu J, Footman A, Qin Y, BRCA1 Mutation Leads to Deregulated Ubc9 Levels which Triggers Proliferation and Migration of Patient-Derived High Grade Serous Ovarian Cancer and Triple Negative Breast Cancer Cells. Int J Chronic Dis Ther. 2016; 2(3): 31–38. 28164176 PMC5287352

[28] Driscoll JJ, Pelluru D, Lefkimmiatis K, The sumoylation pathway is dysregulated in multiple myeloma and is associated with adverse patient outcome. Blood. 2010; 115(14): 2827–2834. doi: 10.1182/blood-2009-03-211045 19965618 PMC2854429

[29] Mayer IA, Abramson VG, Lehmann BD, Pietenpol JA. New strategies for triple-negative breast cancer--deciphering the heterogeneity. Clin Cancer Res. 20(4): 782–790. doi: 10.1158/1078-0432.CCR-13-0583 PMC3962777 24536073

[30] Collett K, Stefansson IM, Eide J, A basal epithelial phenotype is more frequent in interval breast cancers compared with screen detected tumors. Cancer Epidemiol Biomarkers Prev. 2005; 14(5): 1108–1112. doi: 10.1158/1055-9965.EPI-04-0394 15894660

[31] Pettersson A, Graff RE, Ursin G, Mammographic density phenotypes and risk of breast cancer: a meta-analysis. J Natl Cancer Inst. 2014; 106(5). doi: 10.1093/jnci/dju078 PMC4568991 24816206

[32] Sherratt MJ, McConnell JC, Streuli CH. Raised mammographic density: causative mechanisms and biological consequences. Breast Cancer Res. 2016; 18(1): 45. doi: 10.1186/s13058-016-0701-9 27142210 PMC4855337

[33] Green VL. Mammographic Breast Density and Breast Cancer Risk: Implications of the Breast Density Legislation for Health Care Practitioners. Clin Obstet Gynecol. 2016; 59(2): 419–438. doi: 10.1097/GRF.0000000000000192 26992182

[34] Bertrand KA, Tamimi RM, Scott CG, Mammographic density and risk of breast cancer by age and tumor characteristics. Breast Cancer Res. 2013; 15(6): R104. doi: 10.1186/bcr3570 24188089 PMC3978749

[35] Kerlikowske K, Phipps AI. Breast density influences tumor subtypes and tumor aggressiveness. J Natl Cancer Inst. 2011; 103(15): 1143–1145. doi: 10.1093/jnci/djr263 21795663 PMC3149045

[36] JingSong H, Hong G, Yang J, siRNA-mediated suppression of collagen type iv alpha 2 (COL4A2) mRNA inhibits triple-negative breast cancer cell proliferation and migration. Oncotarget. 2017; 8(2): 2585–2593. doi: 10.18632/oncotarget.13716 27906681 PMC5356825

[37] Li F, Li X, Kou L, Li Y, Meng F, Ma F. SUMO-conjugating enzyme UBC9 promotes proliferation and migration of fibroblast-like synoviocytes in rheumatoid arthritis. Inflammation. 2014; 37(4): 1134–1141. doi: 10.1007/s10753-014-9837-x 24531852

[38] DeFilippis RA, Chang H, Dumont N, CD36 repression activates a multicellular stromal program shared by high mammographic density and tumor tissues. Cancer Discov. 2012; 2(9): 826–839. doi: 10.1158/2159-8290.CD-12-0107 22777768 PMC3457705

[39] Hartig SM, Bader DA, Abadie KV, Ubc9 Impairs Activation of the Brown Fat Energy Metabolism Program in Human White Adipocytes. Mol Endocrinol. 2015; 29(9): 1320–1333. doi: 10.1210/me.2015-1084 26192107 PMC4552434

[40] Kim S, Jeon M, Lee J, Induction of fibronectin in response to epidermal growth factor is suppressed by silibinin through the inhibition of STAT3 in triple negative breast cancer cells. Oncol Rep. 2014; 32(5): 2230–2236. doi: 10.3892/or.2014.3450 25175149

[41] Revollo JR, Li X. The ways and means that fine tune Sirt1 activity. Trends Biochem Sci. 2013; 38(3): 160–167. doi: 10.1016/j.tibs.2012.12.004 23394938 PMC4102005

[42] Xu Jingyao, Shumate Collin, Qin Yulong A novel Ubc9 -dependent pathway regulates SIRT1- ER-α Axis and BRCA1-associated TNBC lung metastasis. Integr Mol Med. 2017; 4(4): 2–7. doi: 10.15761/IMM.1000298 PMC6655434 31341634

[43] Rifaï K, Judes G, Idrissou M, Dual SIRT1 expression patterns strongly suggests its bivalent role in human breast cancer. Oncotarget. 2017; 8(67): 110922–110930. doi: 10.18632/oncotarget.23006 29340027 PMC5762295

[44] Li H, Sekine M, Tung N, Avraham HK. Wild-type BRCA1, but not mutated BRCA1, regulates the expression of the nuclear form of beta-catenin. Mol Cancer Res. 2010; 8(3): 407–420. doi: 10.1158/1541-7786.MCR-09-0403 20215423 PMC2867250

[45] Huang HJ, Zhou LL, Fu WJ, β-catenin SUMOylation is involved in the dysregulated proliferation of myeloma cells. Am J Cancer Res. 2014; 5(1): 309–320. 25628940 PMC4300696

[46] Wolff AC, Hammond MEH, Allison KH, Human Epidermal Growth Factor Receptor 2 Testing in Breast Cancer. American Society of Clinical Oncology/College of American Pathologists Clinical Practice Guideline Focused Update. Arch Pathol Lab Med. 2018; 142(11): 1364–1382. doi: 10.5858/arpa.2018-0902-SA 29846104

